# The altered functional connectivity density related to cognitive impairment in alcoholics

**DOI:** 10.3389/fpsyg.2022.973654

**Published:** 2022-08-25

**Authors:** Ranran Duan, Yanfei Li, Lijun Jing, Tian Zhang, Yaobing Yao, Zhe Gong, Yingzhe Shao, Yajun Song, Weijian Wang, Yong Zhang, Jingliang Cheng, Xiaofeng Zhu, Ying Peng, Yanjie Jia

**Affiliations:** ^1^Department of Neurology, The First Affiliated Hospital of Zhengzhou University, Zhengzhou, China; ^2^Department of Magnetic Resonance Imaging, The First Affiliated Hospital of Zhengzhou University, Zhengzhou, China; ^3^Engineering Technology Research Center for Detection and Application of Brain Function of Henan Province, The First Affiliated Hospital of Zhengzhou University, Zhengzhou, China; ^4^Key Laboratory of Magnetic Resonance and Brain Function of Henan Province, The First Affiliated Hospital of Zhengzhou University, Zhengzhou, China; ^5^Key Laboratory of Brain Function and Cognitive Magnetic Resonance Imaging of Zhengzhou, The First Affiliated Hospital of Zhengzhou University, Zhengzhou, China; ^6^Mudanjiang Medical University, Mudanjiang, China; ^7^Department of Neurology, Sun Yat-sen Memorial Hospital, Sun Yat-sen University, Guangzhou, China

**Keywords:** alcohol use disorder (AUD), alcohol-related cognitive impairment (ARCI), functional MRI, functional connectivity density (FCD), global functional connectivity density (gFCD), local functional connectivity density (lFCD), long-range functional connectivity density (lrFCD)

## Abstract

Alcohol use disorder (AUD) is one of the most common substance use disorders contributing to both behavioral and cognitive impairments in patients with AUD. Recent neuroimaging studies point out that AUD is a typical disorder featured by altered functional connectivity. However, the details about how voxel-wise functional coordination remain unknown. Here, we adopted a newly proposed method named functional connectivity density (FCD) to depict altered voxel-wise functional coordination in AUD. The novel functional imaging technique, FCD, provides a comprehensive analytical method for brain's “scale-free” networks. We applied resting-state functional MRI (rs-fMRI) toward subjects to obtain their FCD, including global FCD (gFCD), local FCD (lFCD), and long-range FCD (lrFCD). Sixty-one patients with AUD and 29 healthy controls (HC) were recruited, and patients with AUD were further divided into alcohol-related cognitive impairment group (ARCI, n = 11) and non-cognitive impairment group (AUD-NCI, n = 50). All subjects were asked to stay stationary during the scan in order to calculate the resting-state gFCD, lFCD, and lrFCD values, and further investigate the abnormal connectivity alterations among AUD-NCI, ARCI, and HC. Compared to HC, both AUD groups exhibited significantly altered gFCD in the left inferior occipital lobe, left calcarine, altered lFCD in right lingual, and altered lrFCD in ventromedial frontal gyrus (VMPFC). It is notable that gFCD of the ARCI group was found to be significantly deviated from AUD-NCI and HC in left medial frontal gyrus, which changes probably contributed by the impairment in cognition. In addition, no significant differences in gFCD were found between ARCI and HC in left parahippocampal, while ARCI and HC were profoundly deviated from AUD-NCI, possibly reflecting a compensation of cognition impairment. Further analysis showed that within patients with AUD, gFCD values in left medial frontal gyrus are negatively correlated with MMSE scores, while lFCD values in left inferior occipital lobe are positively related to ADS scores. In conclusion, patients with AUD exhibited significantly altered functional connectivity patterns mainly in several left hemisphere brain regions, while patients with AUD with or without cognitive impairment also demonstrated intergroup FCD differences which correlated with symptom severity, and patients with AUD cognitive impairment would suffer less severe alcohol dependence. This difference in symptom severity probably served as a compensation for cognitive impairment, suggesting a difference in pathological pathways. These findings assisted future AUD studies by providing insight into possible pathological mechanisms.

## Introduction

Alcohol use disorder (AUD) is a progressive chronic disease, and it is considered a major public health problem in the world (Kranzler and Soyka, 2018; Flook et al., 2020). With heightened reactivity to alcohol-associated stimuli being hallmarks of AUD (Oberlin et al., 2020), patients with AUD are suffered from compulsive alcohol drinking and even impaired cognitive functions (Stavro et al., 2013; Duan et al., 2022). During the past few decades, many researchers have applied functional magnetic resonance imaging (fMRI) to assess the functional connectivity strength, which represents the temporal association of spontaneous brain activity alterations between two selected brain regions (Biswal et al., 1995), with an altered pattern in functional connectivity was considered to be associated with impaired brain functions.

Many efforts have been devoted to the exploration of functional connectivity, and significantly altered functional connectivity patterns in patients with AUD were found by many researchers. For example, Han et al. have reported in a resting-state fMRI study that alcohol addicts showed a wide range of abnormal functional connections including the frontal lobe, anterior cingulate gyrus, bilateral insula, thalamus, precuneus, left caudate nucleus, and some temporal and occipital lobes (Han et al., 2021). Compared with the healthy control (HC), the AUD group decreased in the right anterior cuneiform lobe and left cerebellar peduncle II area, but increased in the left posterior cingulate gyrus, right middle temporal gyrus, right superior temporal gyrus, and right anterior cuneiform lobe area, indicating an abnormal default mode network function (DMN) (Khan et al., 2021a), where was later found to have increased functional connectivity in patients with AUD (Yang et al., 2021). In addition, patients with AUD also exhibited enhanced functional connections in the orbitofrontal cortex network, left executive control network, amygdala striatum network, executive control network, and visual input network, possibly severed as a neural compensation (Müller-Oehring et al., 2015). In contrast, the functional connectivity of the basal ganglia network and primary visual network was significantly reduced in alcohol-addicted patients, and the functional connectivity intensity of the executive control network was negatively correlated with the degree of alcohol abuse (Weiland et al., 2014). However, most of the previous studies that investigated the abnormal pattern in AUD's functional connectivity focused on hypothesis-driven methods based on region of interest (ROI) or independent component analysis (ICA), which only explored limited specific networks seed. Therefore, the complete picture of voxel-wise functional coordination remains unclear.

Recently, resting-state functional connectivity density (rsFCD) was proposed to provide a comprehensive analytical method for brain's “scale-free” networks by comparing differences in functional connectivity between the whole brain and the given voxel in a high spatial resolution during the past few years, and FCD mapping has proven to be instructive in diagnosing and providing supplementary evidence for symptom improvement of several psychiatric disorders such as depression and schizophrenia (Mao et al., 2021; Wang et al., 2021; Yang et al., 2021), and there were also studies investigating that smoking and internet addiction had applied FCD to demonstrate the link between the aberrance in brain functional connectivity and addictive behaviors (Zhao et al., 2019; Wang et al., 2020). Although several FCD research were conducted on AUD, these studies focused on the early diagnosis and assessment (Holla et al., 2017; Shokri-Kojori et al., 2017) instead of probing into the correlation of pathological changes in functional connectivity with the development of AUD.

Previous mechanistic AUD studies mostly utilized seed-based strategies which are highly dependent on the prior selection (Tomasi and Volkow, 2010). Recently, novel methods such as functional connectivity density (FCD) are proposed to overcome this weakness. FCD is a pattern that allows researchers to assess whole-brain functional brain connectivity at the voxel level. At the same time, FCD can also reflect the characteristics of spontaneous neural activity, revealing the functional relationship between different brain regions. The higher the FCD value of a particular voxel, the more connected it is to other brain voxels, suggesting that these voxels play a greater role in information processing. FCD mapping is a graph-based network organization measurement that reflects the number of transient functional connections between a region and other parts of the brain in the connectivity matrix for the whole brain. Thus, it is possible to assess the impact of a node on the whole brain and integrate information across functionally isolated brain regions. As a data-driven method based on voxel analysis, FCD is more easy and independent from any prior assumption. FCD overcomes the limitation of function connection based on seed point and describes information processing in a certain area. It focuses on the functional connection strength of the voxel (i.e., the “hub” of the highway in the brain network) rather than the specific connection path, thus indicating the distribution of core nodes for functional connections (Tomasi and Volkow, 2011). Higher FCD values of a region indicate a tighter and larger connection with other brain voxels, suggesting the importance of this specific brain region during information processing. In addition, FCDC mapping is able to measure local FCD values as well as global FCD values, giving more details on the functional connections of a specific brain region. Therefore, FCD could provide information about the crucial network hubs when the brain undergoes pathological changes in functional and structural, thus is instructive for revealing the correlation between altered FCD values and the pathological mechanisms of AUD.

In the current study, we applied rs-fMRI to compare the functional connectivity alterations in patients with AUD using FCD mapping, and investigated its association with symptom severity. Sixty-one patients with AUD and 29 matched healthy controls were recruited, with AUD being divided into alcohol-related cognitive impairment (ARCI, n = 11) or AUD with non-cognitive impairment group (AUD-NCI, n = 50) groups.

## Materials and methods

### Sample

This present study was approved by the Medical Ethics Committee of the First Affiliated Hospital of Zhengzhou University and complied with the declaration of Helsinki, as revised in 2008, and all participants provided informed written consent before completing the survey.

Individuals with AUD were recruited from a variety of sources including inpatient ward, internet posting, and advertisements. The control participants were volunteers from the local communities.

Inclusion criteria were: (1) meeting the DSM-5 criteria for AUD, based on the clinical assessment of the principal investigator; (2) drinking on average more than 14 units of alcohol per week, according to the U.K. Chief Medical Officers (Stautz et al., 2017); (3) Clinical Institute Withdrawal Assessment-advanced Revised (CIWA-Ar) <9; (4) could understand and consent to study procedures.

Primary exclusion criteria for both AUD and healthy controls (HCs) were: (1) having a history of addictive (alcohol excepted), psychiatric, neurological, or physical disorder that could influence brain morphology; (2) having contraindications for magnetic resonance imaging (MRI); (3) reported currently taking centrally active medications. All subjects included in this study were men.

Demographic and clinical data are shown in Table 1. The sample consisted of 29 HCs and 61 patients with AUD. We measured cognitive impairment using the Mini-Mental State Examination (MMSE) and Montreal Cognitive Assessment (MoCA). All participants ranged in age from 18 to 65. The HCs were screened using the Structured Clinical Interview (SCID)-non-patient edition (First et al., 2002), and the HCs were confirmed not to have any present or previous mental health problems. All subjects were screened using the Alcohol Dependence Scale (ADS), Clinical Institute Withdrawal Assessment-advanced Revised (CIWA-Ar), Visual Analog Scale (VAS), Obsessive Compulsive Drinking Scale (OCDS), Generalized Anxiety Disorder (GAD-7), Patient Health Questionnaire (PHQ-9), and Pittsburgh Sleep Quality Index (PSQI).

**Table 1:** Demographics of subjects (one-way ANOVA).

	**AUD-NCI (n = 50)**	**ARCI (n = 11)**	**HC (n = 29)**	***p***
Age (mean ± SD), y	46.88 ± 8.95	49.45 ± 7.95	46.44 ± 10.83	0.6700
Years of schooling (mean ± SD), y	10.42 ± 1.98	10.82 ± 2.23	10.67 ± 3.00	0.8372
High (mean ± SD), cm	172.40 ± 4.76	170.8 ± 4.55	175.3 ± 5.48	0.1309
Weight (mean ± SD), Kg	74.56 ± 9.99	77.39 ± 10.85	77.67 ± 4.70	0.5491
Time of alcohol drinking (mean ± SD), y	24.58 ± 10.85	22.30 ± 8.00	0.53 ± 0.39	<0.001
Pure alcohol (total, mean ± SD), g	346,522.98 ± 40,938.76	374,521.66 ± 39,845.86	765.99 ± 187.17	<0.001
ADS (mean ± SD)	13.62 ± 6.80	11.18 ± 8.75	0.44 ± 0.73	<0.001
CIWA-Ar (mean ± SD)	8.42 ± 6.76	4.82 ± 4.49	0 ± 0	<0.001
OCDS (mean ± SD)	14.94 ± 8.67	13.09 ± 7.82	0.22 ± 0.67	<0.001
VAS (mean ± SD)	2.16 ± 2.46	2.45 ± 2.77	0.22 ± 0.67	<0.001
MoCA (mean ± SD)	27.52 ± 1.39	18.64 ± 3.53	29.00 ± 1.00	<0.001
PSQI (mean ± SD)	6.44 ± 3.10	5.36 ± 4.32	6.0 ± 2.50	0.5992
GAD-7 (mean ± SD)	4.48 ± 4.53	3.73 ± 6.36	5.44 ± 2.50	0.4657
PHQ-9 (mean ± SD)	5.92 ± 5.22	5.90 ± 7.97	3.56 ± 2.46	0.4751
MMSE (mean ± SD)	27.40 ± 1.70	19.91 ± 3.02	29.44 ± 0.73	<0.001

*ADS, Alcohol Dependence Scale; CIWA-Ar, Clinical Institute Withdrawal Assessment-advanced Revised; OCDS, Obsessive Compulsive Drinking Scale; VAS, Visual Analog Scale; MoCA, Montreal Cognitive Assessment; PSQI, Pittsburgh Sleep Quality Index; GAD-7, Generalized Anxiety Disorder; PHQ-9, Patient Health Questionnaire; MMSE, Mini-Mental State Examination; AUD-NCI, Alcohol Use Disorder-Non-cognitive Impairment Group; ARCI, Alcohol-Related Cognitive Impairment Group; HC, Healthy Controls*.

Patients with AUD recruited for our study were further divided into alcohol-related cognitive impairment group (ARCI) (MMSE <24 and MoCA <26) and non-cognitive impairment group (AUD-NCI) (MMSE≥24 and MoCA ≥26) by testing MMSE and MoCA scales (Nasreddine et al., 2005; Chan et al., 2017).

### Neuroimaging data acquisition

All MRI images were obtained in SIEMENS 3.0T scanner (MAGNETOM Prisma, SIEMENS, Germany) with a 16-channel head coil at the First Affiliated Hospital of Zhengzhou University. All subjects were requested to keep their eyes closed, and foam padding and earplugs were used to control participants' head movements and reduce noise. At the end of scanning, subjects were also asked if they had fallen asleep during scanning. The rs-fMRI data were obtained using the following parameters: TR = 1,000 ms, TE = 30 ms, field of view 220^*^220 mm^2^, slice thickness 2.2 mm, slice gap 0.4 mm, flip angle 70°, and voxel size 2.0 × 2.0 × 2.2 mm^3^, with 52 slices and 400 dynamics. The slices aligned along the AC-PC line were acquired with a total scan time of 360 s.

### Data preprocessing

Functional images were preprocessed following the pipeline of the Data Processing Assistant for Resting-State fMRI package (http://www.restfmri.net) (Han et al., 2020). The main steps included the removal of the first 10 volumes, slice timing correction, and realignment. Subjects would be excluded if the translational and rotational displacement exceeded 3.0 mm or 3.0°across the entire scan. Images were resampled to 3 mm^3^ and normalized to the standard EPI template. Smoothing with 6 mm^3^ full-width at half maximum Gaussian kernel, detrend, filtered with band-pass (0.01–0.1 Hz), and regression of nuisance covariates including Friston 24 motion parameters (Satterthwaite et al., 2012), white matter signal, and cerebrospinal fluid signal. Finally, to further excluded the effect of head motion, scrubbing with cubic spline interpolation was used (Power et al., 2012). The “bad” points were identified with a threshold of frame displacement larger than 0.5 mm as well as one-forward and two-back neighbors (Power et al., 2012). To further exclude the effect of head motion on our results, we calculated the mean frame-wise displacement (FD) for each subject (Wang et al., 2017, 2019; Han et al., 2020).

### Calculation of functional connectivity density

As done in the previous study, local, long-range, and global FCD maps were calculated (Tomasi and Volkow, 2010). The global FCD of a voxel was defined as the number of significant functional connections between it with other voxels in the gray matter. The local FCD was defined as the size of a continuous cluster of spatially connected voxels that were significantly correlated with a given voxel (Tomasi and Volkow, 2012). The long-range FCD was obtained by global FCD minus local FCD (Tomasi and Volkow, 2012). A functional connection (Pearson's correlation) was considered significant of its *p* value <0.05 (Bonferroni corrected for voxels across gray matter). FCD maps were further transformed to z scores by subtracting the mean value and dividing by the standard deviation across gray matter voxels in the brain (Liao et al., 2013). The following statistical analysis was done based on the z-map of FCD.

### Statistical analysis

One-way ANOVA was used to compare (global, local, or long-range) FCD maps among the three groups, respectively. In this procedure, age and mean FD were included as covariates. Results reported in this study were corrected for multiple comparison [voxel-wise *p* < 0.001, cluster-level *p* < 0.05; Gaussian random field (GRF) corrected]. Then, we extracted mean FCD values presenting significant differences among the three groups by averaging each peak coordinate with a spherical radius of 6 mm. The mean FCD values were compared with each pair of groups by using a two-tailed two-sample t-test.

### Association with symptom severity

To investigate the correlation between altered FCD values and symptom severity, Pearson's correlations between these altered FCD values (extracted before) and scores of clinical scales were calculated.

## Results

### Clinical demographics

The clinical demographics of the subjects are shown in Table 1. The sample consisted of 29 HCs and 61 patients with AUD. Among the AUD subjects, 11 subjects suffered from cognitive impairment. The three groups presented no significant difference in age, sex, years of schooling, high, and weight. The MoCA and MMSE scores were lower in the ARCI group than in the HCs and AUD-NCI groups (*p* < 0.001), and ADS and OCDs scores were higher in the ARCI and AUD-NCI groups than in the HCs group (*p* < 0.001). The details are shown in Table 1.

### Altered FCD in AUD

**Figure 1:**
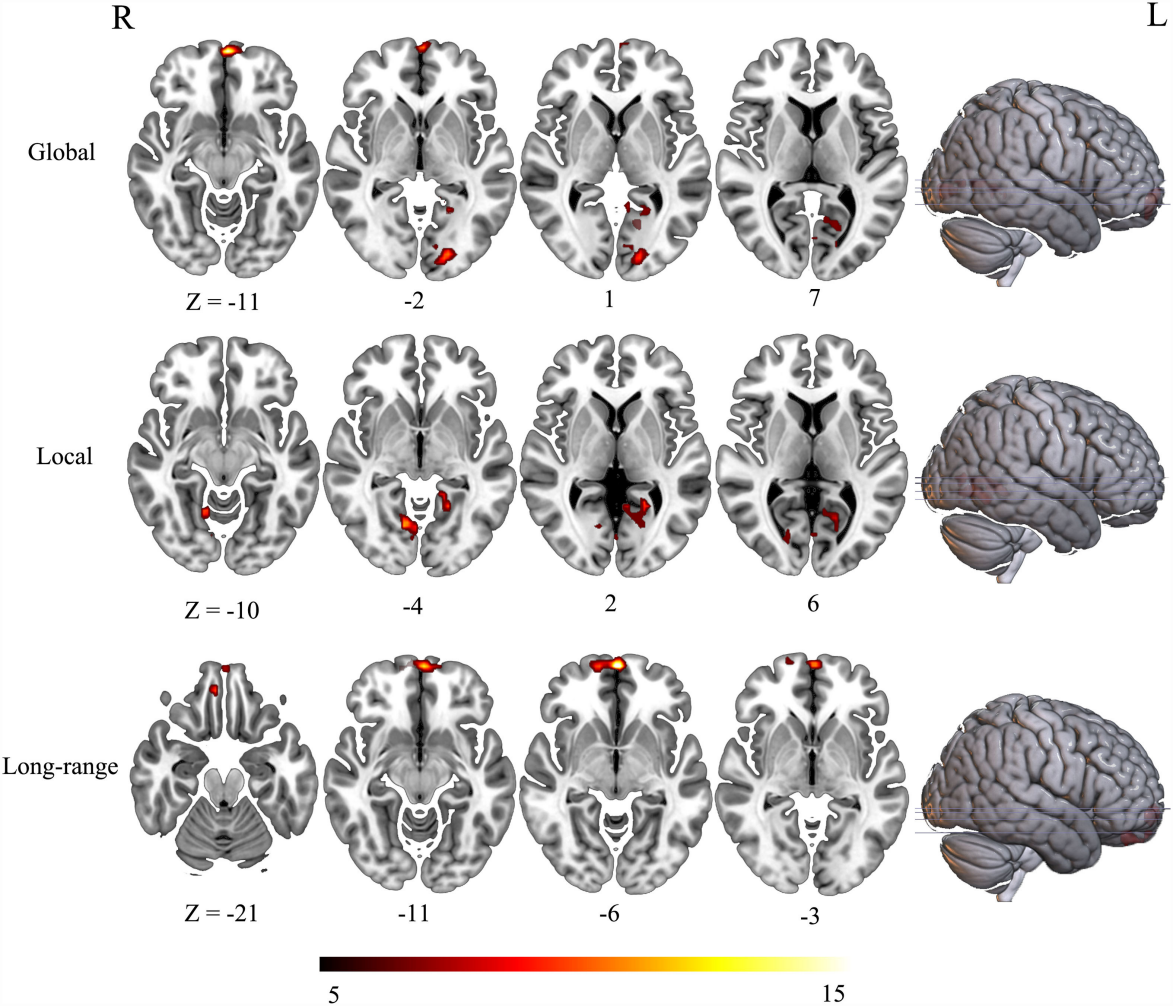
Altered FCD among the three groups (ANOVA results).

The three groups presented significant difference in FCD maps (voxel-wise *p* < 0.001, cluster-level *p* < 0.05; GRF corrected). Specially, the main effect of global FCD was located in brain regions including left medial frontal gyrus, left parahippocampal gyrus, left inferior occipital lobe, and left calcarine (Figure 1). As for local FCD, these three groups exhibited a significant difference in brain regions such as left parahippocampal gyrus and left lingual (Figure 1). In addition, left VMPFC presented differences in long-range FCD among these three groups (Table 2).

**Table 2:** ANOVA results of FCD.

**FCD**	**Clusters**	**Voxels**	**Including regions**	**Peak MNI (x, y, z)**	**F**
Global	1	59	Medial frontal gyrus	0, 66, −6	20.60
	2	54	Left inferior occipital lobe	−21, −87, −3	11.32
	3	43	Left calcarine	−18, −66, 3	10.27
			Left parahippocampal gyrus		
Local	1	56	Left parahippocampal gyrus	−21, −51, 0	13.20
			Left lingual		
			Left calcarine		
	2	52	Right lingual	12, −69, −3	12.38
Long-range	1	73	Left medial frontal gyrus	0, 63, −6	18.86
			Left superior frontal gyrus		
	2	37	VMPFC	6, 45, −24	11.64

**Figure 2:**
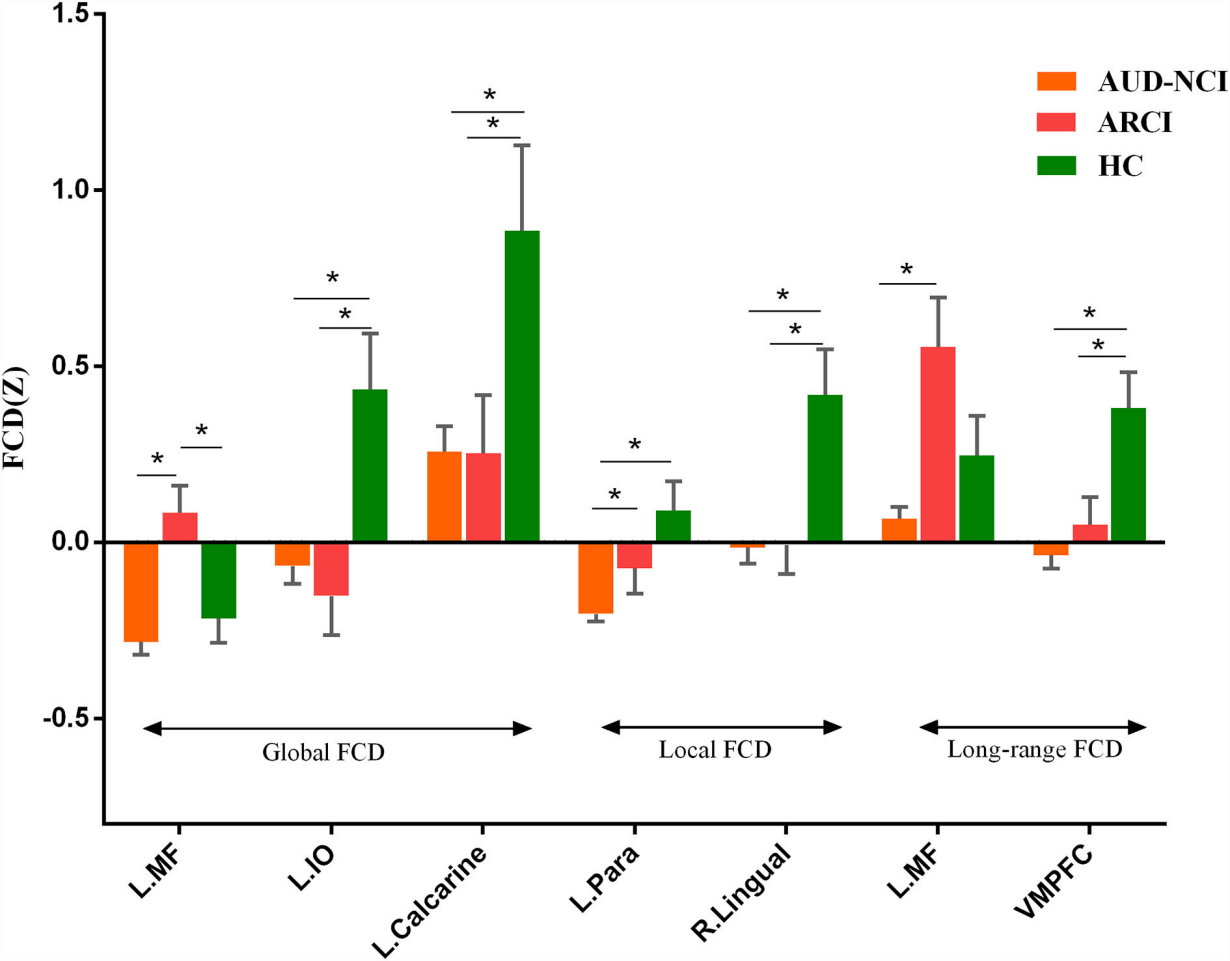
*Post-hoc* analysis results. MF, medial frontal gyrus; IO, inferior occipital lobe; Para, parahippocampal gyrus; VMPFC, ventromedial frontal gyrus.

To further determine the details about FCD aberrance. We extracted the mean FCD values by averaging Z scores of each peak coordinate with a spherical radius of 6 mm. Then we compared them between pair of groups by using the two-tailed two-sample t-test. The peak MNI coordinates and the results are shown in Figure 2; Table 3.

**Table 3:** *Post-hoc* analysis results.

**FCD**	**Regions**	**MNI (x, y, z)**	**T(AUD-NCI vs. ARCI)**	**T(AUD-NCI vs. HC)**	**T(ARCI vs. HC)**
Global	Medial frontal gyrus	0, 66, −6	−4.34^*^	−0.74	2.88^*^
	Left inferior occipital lobe	−21, −87, −3	0.71	−3.68^*^	−3.11^*^
	Calcarine	0, −75, 6	0.03	−3.18^*^	−2.23^*^
Local	Parahippocampal Gyrus	−21, −51, 0	−2.32^*^	−4.89^*^	−1.51
	Right Lingual	12, −69, −3	−0.08	−3.64^*^	−2.88^*^
Long-range	Medial frontal gyrus	0, 63, −6	−5.07^*^	−1.97	1.64
	VMPFC	6, 45, −24	−0.99	−4.28^*^	−2.62^*^

The “^*^” meant the difference was significant (p <0.05).

*AUD-NCI, alcohol use disorder-non-cognitive impairment group; ARCI, alcohol-related cognitive impairment group; HC, healthy controls*.

### Association with clinical symptoms

**Figure 3:**
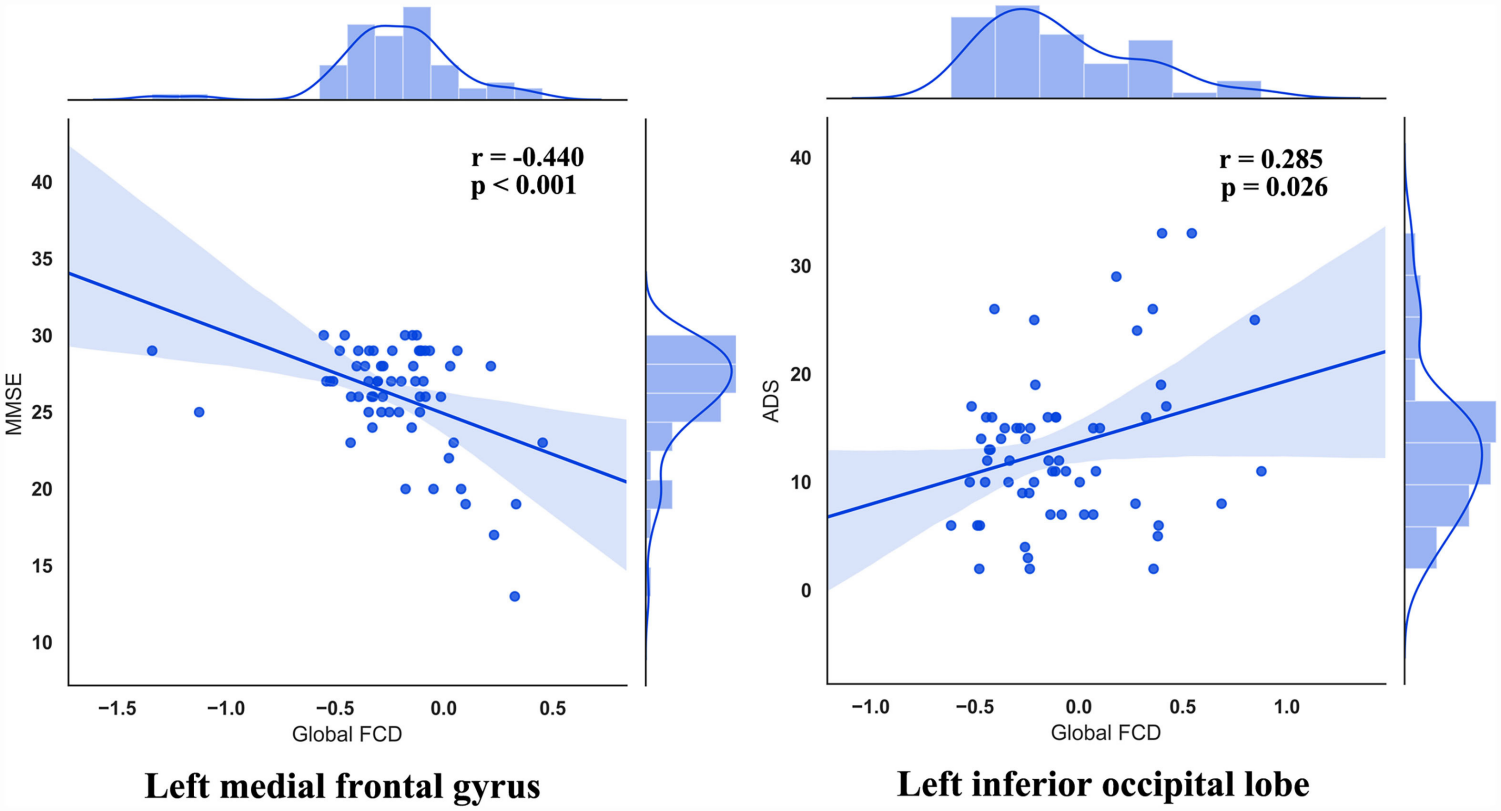
The association between altered FCD with clinical symptoms.

We found a significant association between altered FCD with clinical symptoms (Figure 3). Specifically, the global FCD values in left medial frontal gyrus and that in left inferior occipital lobe were correlated with MMSE (*r* = −0.440, uncorrected *p* < 0.001) and ADS (*r* = 0.285, uncorrected *p* = 0.026), respectively.

## Discussion

In the current study, we explored local, long-range, and global FCD maps which come from healthy controls and patients with AUD, and we extracted mean FCD values presenting significant differences among the three groups. Besides, we found that the main effect of global FCD was located in brain regions and local FCD of the three groups exhibited significant differences in brain regions. Through statistical analysis of relevant data, we found a significant association between altered FCD with clinical symptoms. Specifically, the global FCD values in left medial frontal gyrus and that in left inferior occipital lobe were correlated with MMSE and ADS, respectively, and long-range FCD values in left medial frontal gyrus were correlated with MMSE. This suggests that alternations of FCD might be associated with AUD.

Our study finds that the left medial frontal gyrus has significantly deviated among HC, ARCI, and AUD-NCI in gFCD. This change in gFCD possibly stems from a significantly lower density of medial frontal gyrus in patients with AUD found in a previous finding (Mechtcheriakov et al., 2007). Significant differences in left parahippocampal gyrus in gFCD and lFCD are also discovered. The aberrant pattern in lFCD reveals an impaired within-region activity, supported by earlier research findings that AUD subjects exhibit a parahippocampal gyrus volume shrinkage and a regional cerebral blood flow decrease (Suzuki et al., 2010), together with a prominent loss of white matter (Jang et al., 2007). In addition to anatomy, parahippocampal gyrus is also a crucial node linking the default mode network (DMN) and medial temporal lobe (Zhang and Volkow, 2019). In patients with AUD, the effective connectivity within DMN is lower than HC (Khan et al., 2021b), thus impaired parahippocampal gyrus might associate with this contraction in connectivity. The current study also recognizes a significant difference in VMPFC's long-range FCD, representing disrupted functional connectivity between VMPFC and other brain regions. This change accords with general ideas that VMPFC is blunt toward stress in patients with AUD (Seo and Sinha, 2015; Hwang et al., 2021). Moreover, left inferior occipital lobe and left calcarine also present significant gFCD differences, and right lingual as well regarding lFCD. These three regions contribute to visual information processing, while visual processing deficits are a common symptom for patients with AUD (Bagga et al., 2014). The volume of gray matter in the left occipital lobe in AUD subjects has been found significantly decreased, which might be a causal factor of impairment (Zou et al., 2018).

The *post-hoc* analysis results among HC, ARCI, and AUD-NCI reveal more details. Left inferior occipital lobe, calcarine, right lingual, and VMPFC all exhibit significant differences between HC and both AUD groups, but no significance between AUD groups. As for left inferior occipital lobe and calcarine, AUD groups show significant decreases in gFCD than HC. The right lingual's lFCD and VMPFC's long-range FCD present a similar pattern with higher activity in HC. Since left inferior occipital lobe, calcarine, and right lingual all play roles in visual information processing, this indifference might imply a visual processing deficit in AUD regardless of cognitive state. The decreased long-range FCD in VMPFC is consistent with the consensus that VMPFC is hypo-activated in patients with AUD (Seo et al., 2016). Moreover, this decrease in VMPFC connectivity can also be found in other substance use disorders (SUD) like cocaine addiction (Miller, 2000). It is intriguing to posit that this decreased activity in VMPFC might be a prevalent phenomenon in SUD. In terms of medial frontal gyrus, there is no significance between AUD-NCI and HC, but ARCI's gFCD is significantly higher compared to both AUD-NCI and HC. Medial frontal gyrus participates in cognition, assisting high-level executive functions and decision-related process (Talati and Hirsch, 2005). This increased connectivity is in consonance with an earlier study on mild cognitive impairment (MCI), which proposed that HC contains a negative functional connectivity peak in medial frontal gyrus, while patients with MCI contains a peak in opposite direction (Bokde et al., 2006). However, it is unusual that significance exists in NCI-ARCI and NCI-HC for parahippocampal gyrus, probably suggesting a compromise between parahippocampal gyrus function and cognitive impairment in the AUD population, namely AUD without CI might suffer more severe disruption in parahippocampal gyrus activity than ARCI.

The results of Pearson's correlations showed that gFCD values in the left medial frontal gyrus were negatively correlated with MMSE scores in AUD groups, namely a higher gFCD indicates a more severe cognitive impairment. The current study clarifies that the ARCI group has higher gFCD values in left medial frontal gyrus than the AUD-NCI group, consistent with the division of AUD subgroups. This abnormally increased activity in left medial frontal gyrus is not limited to patients with AUD but also exists in patients with Alzheimer's and MCI during working memory tasks (Yetkin et al., 2006), and probably stem from an increased fiber density between medial frontal gyrus and superior frontal gyrus (Chumin et al., 2019; Duan et al., 2022). However, the AUD-NCI group has significantly smaller gFCD values than the ARCI group, in consensus with the previous finding that the gray matter volume in left medial frontal gyrus is decreased in patients with AUD (Yang et al., 2016). The contradiction between AUD-NCI and ARCI groups possibly suggest different pathological mechanisms. The variance between the two AUD groups was also seen in the left inferior occipital lobe. The Pearson's correlation demonstrates that left inferior occipital lobe gFCD values are positively correlated with ADS scores, meaning that the AUD-NCI group with lower gFCD values has a lighter dependence on alcohol. According to Yang et al. (2016), the shrinkage in the gray matter volume of medial frontal gyrus is positively associated with heavier alcohol consumption. Together with the results of the current study, it is intriguing to hypothesize that although ARCI has more severe damage to cognitive abilities, as a compensation, patients with ARCI have less alcohol dependence and hence less impairment in the left medial frontal gyrus gray matter. In addition, although we did not take the ADS score of HC, HC has the highest gFCD in the left inferior occipital lobe, which means the correlation between ADS and gFCD in the left inferior occipital lobe cannot fit in HC. These alterations in mechanisms between HC and AUD indicate an unseen functional role played by the left inferior occipital lobe, which needs further explorations in future studies.

Nevertheless, some limitations still exist in the current study. First of all, the sample size of the current study is not adequate, and future studies with an enlarged subjects number are required to assess whether the results are reproducible in a large clinical population. In addition, the significant difference between subject groups damaged the reliability of results and needs to be balanced in future studies for unbiased results. It is also notable that we did not identify whether the altered FCD pattern found in patients with AUD is a consequence of alcohol addiction or indicated as an addiction risk factor. Future studies that explore the causal relationships between this abnormal FCD pattern and AUD are required to elucidate the role of altered FCD. Furthermore, longitude studies that explore the correlation between the deviation of FCD from HC and the length of the course of AUD, and dynamic FCD under tasks should be involved in future investigation instead of stationary FCD in order to promote the validity in real-life activities.

## Conclusion

In conclusion, in this FCD study, patients with AUD were found to significantly alter the rs FCD in patients with AUD with or without cognitive impairment compared to healthy controls patients with AUD suffering from a significant decrease in whole-brain functional connectivity in left medial frontal gyrus, the left parahippocampal gyrus, the left inferior occipital lobe, and the left cerebellum. Besides, the functional connectivities within the left parahippocampal gyrus and the right lingual gyrus are damaged in patients with AUD as well. In addition, these alterations are associated with clinical symptoms of patients with AUD, with more severely impaired brain regions contributing to higher alcohol dependency or lower cognitive ability. These abnormal patterns in FCD provide evidence for the potential neurological mechanisms contributing to AUD, indicating that different AUD subgroups have distinct pathological pathways, and assist further understanding toward the brain network activities in patients with AUD.

## Data availability statement

The raw data supporting the conclusions of this article will be made available by the authors, without undue reservation.

## Ethics statement

This present study was approved by the Medical Ethics Committee of the First Affiliated Hospital of Zhengzhou University and complied with the declaration of Helsinki, as revised in 2008, and all participants provided informed written consent before completing the survey.

## Author contributions

YJ designed and supervised all aspects of the study, namely, the fMRI protocol, fMRI data processing, analysis and drafted the manuscript, and subsequent revisions. RD conducted all the fMRI data analysis and drafted the manuscript, created all tables and figures, and assisted with manuscript preparation and revision. YL, LJ, ZG, YY, YSh, and YSo assisted in data analysis and revised the manuscript. WW, YZ, JC, XZ, and YP provided guidance on study design and analysis, assisted with the fMRI acquisition protocol, and reviewed and revised the manuscript. All authors read and approved the final manuscript.

## Funding

This research was supported by the National Key R&D Program of China (2018YFC1314400 and 2018YFC1314403).

## Conflict of interest

The authors declare that the research was conducted in the absence of any commercial or financial relationships that could be construed as a potential conflict of interest.

## Publisher's note

All claims expressed in this article are solely those of the authors and do not necessarily represent those of their affiliated organizations, or those of the publisher, the editors and the reviewers. Any product that may be evaluated in this article, or claim that may be made by its manufacturer, is not guaranteed or endorsed by the publisher.

## Abbreviations

AUD: alcohol use disorder
FCD: functional connectivity density
rs-fMRI: resting-state functional MRI
gFCD: global FCD
lFCD: local FCD
lrFCD: long-range FCD
HC: healthy controls
ARCI: alcohol-related cognitive impairment group
AUD-NCI: non-cognitive impairment group
DMN: default mode network function
ROI: region of interest
ICA: independent component analysis
rsFCD: resting-state functional connectivity density
CIWA-Ar: Clinical Institute Withdrawal Assessment-advanced Revised
MMSE: Mini-Mental State Examination
MoCA: Montreal Cognitive Assessment
SCID: Structured Clinical Interview
ADS: Alcohol Dependence Scale
VAS: Visual Analog Scale
OCDS: Obsessive Compulsive Drinking Scale
GAD-7: Generalized Anxiety Disorder
PHQ-9: Patient Health Questionnaire
PSQI: Pittsburgh Sleep Quality Index
GRF: Gaussian random field
SUD: substance use disorders
MCI: mild cognitive impairment.
